# A Modified Quadrature Birdcage Coil Incorporated With a Curved Feature for *In Ovo* MR Imaging

**DOI:** 10.1109/OJEMB.2024.3420231

**Published:** 2024-06-28

**Authors:** Chang-Hoon Choi, Maximilian Bruch, Suk-Min Hong, Sandra Krause, Carina Stegmayr, Stefan Schwan, Wieland A. Worthoff, Jörg Felder, N. Jon Shah

**Affiliations:** Institute of Neuroscience and Medicine - 4 (INM-4)Forschungszentrum Jülich (FZJ) 28334 52425 Jülich Germany; Aachen University of Applied Sciences 240098 52428 Jülich Germany; INM-4FZJ 28334 52425 Jülich Germany; INM-4FZJ 28334 52425 Jülich Germany; RWTH University 234344 52062 Aachen Germany; INM-4 and INM-11FZJ 28334 52425 Jülich Germany; JARA - BRAIN - Translational Medicine Aachen Germany; Department of NeurologyRWTH Aachen University 9165 52062 Aachen Germany

**Keywords:** Chick embryo, in ovo, MRI, coil, birdcage

## Abstract

*Goal:* This study presents a novel MRI coil design approach explicitly tailored for chick embryo measurements, with the primary objective of improving sensitivity and coverage. *Methods:* The limitations posed by conventional birdcage coils were addressed by introducing a curvature feature into a standard coil. The performance of the modified coil was assessed using EM simulations and experimental evaluations, which were subsequently validated using a 7 T MRI scanner. A comparative analysis was conducted against a standard quadrature low-pass birdcage coil to evaluate key factors. *Results:* The proposed coil demonstrated improved SNR and uniformity, particularly in the proximity of the end-rings. These results were consistent with the findings obtained from the simulations. *Conclusions:* The use of our innovative birdcage coil design holds promise and offers practical potential for *in ovo* studies.

## I. Introduction

With assistance of magnetic resonance imaging (MRI), preclinical rodent-based models have become firmly established and remain the primary choice for fundamental research into various diseases and biomedical applications. Examples include cancer and metastasis developments, as well as the evaluation of newly developed drugs or tumour tracers for precise diagnosis and effective treatment [1], [2], [3]*.* However, conducting the studies using small animals is costly and time-consuming due to regulatory and ethical requirements and the associated complexities. To address these challenges, the use of chick embryos has garnered attention as an alternative to replace traditional animal models. Utilising chick embryos offers simplicity in screening and preparation requirements. This is also coupled with distinct advantages including easy accessibility to their chorioallantoic membranes (CAM), rapid growth, cost-effectiveness, nutritional independence and shared phylogenetic characteristics with mammals [4], [5], [6], [7], [8], [9].

In MRI technology, the birdcage coil has emerged as the preeminent volume radiofrequency (RF) coil [10], [11], [12], owing to its perpendicular RF field (*B*_1_) orientation against the main magnetic field in most superconducting magnets. Since its original development in 1985, various design modifications have been introduced in order to fulfil its specific applications as effectively as possible. These variations include the developments of the so-called four-ring [13], folded four-ring [14], degenerative [15], multi-parallel round leg [16], asymmetric [17], split, half- or open-birdcage [18], [19], [20] and spiral birdcage coils [21]. These inventions have primarily targeted improvements in *B*_1_ homogeneity, signal-to-noise ratio (SNR) and imaging coverage.

In the context of chick embryo measurements using MRI, a standard small animal body birdcage coil is commonly employed [4], [22], [23], [24], [25]. However, this design is mostly intended for objects such as rat bodies. Therefore, the standard coil is not fully tailored for chick embryos, particularly in terms of its size and length. Furthermore, SNR may diminish in areas of the embryo that are located close to the end-rings of the standard coils. While this can simply be compensated for by increasing the length of the birdcage coil, due to the filling factor the quality of the enlarged coil decreases as a function of the increased length and diameter of the coil when the size of the object is identical [12], [26], [27], [28], [29]. Moreover, maintaining consistent imaging orientations is further complicated as the position of the chick embryo changes over time. This means that it may not always be possible to place the embryos or their specific organs, such as their brain, within the sensitive region of the coil or to image those in the same orientation in a single scan, even on consecutive days [25].

This work introduces a new curved approach to coil design that is tailored specifically for chick embryo MR scans. Previous attempts in *in vivo* human or animal experiments have largely aimed to integrate a curved feature solely on one side of the coil [30], [31], [32], [33], mainly due to limited accessibility. By integrating a curved shape into a standard low-pass birdcage coil, SNR is improved while achieving extended coverage. The performance of this modified birdcage coil was scrutinised in electromagnetic (EM) simulation and on the bench. The final validation was conducted using a 7 T MRI scanner. Key evaluation matrices were predominantly compared against a standard quadrature low-pass birdcage coil.

## II. Materials and Methods

### A. EM Simulation

**Fig. 1.**
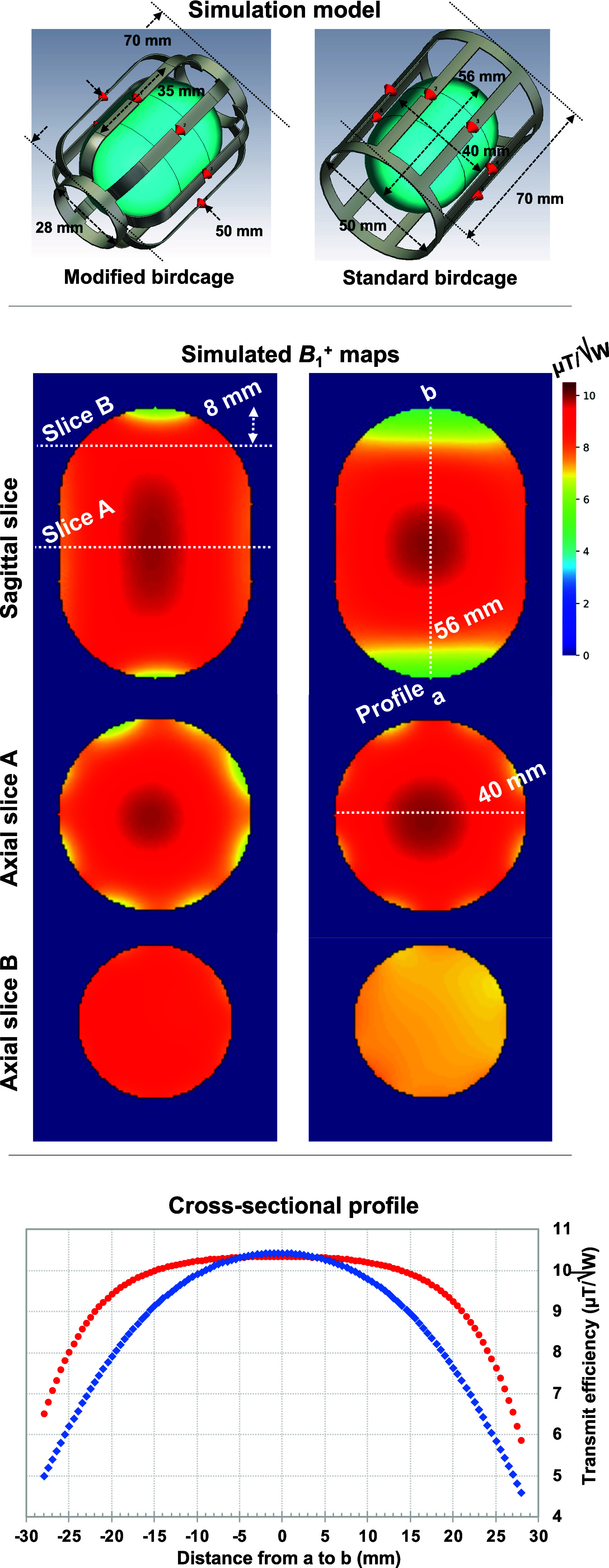
EM simulation models (top) including the coils and the phantom and ${{B}_1}^ + $ field maps in the sagittal and axial planes of the proposed (left) and conventional (right) coils. The horizontal and vertical white dotted lines in the sagittal images indicate the location of the slice cuts for the axial maps and for cross-sectional profiles (bottom), respectively. The white dotted line in the axial slice A image indicates the distance of the phantom, which is 40 mm.

Using the CST Studio Suite (Dassault Systems, Vélizy-Villacoublay, France), a finite integration technique simulation was performed in order to anticipate the characteristics of the newly designed modified birdcage coil. As depicted in Fig. 1, the coils were modelled on the basis of the dimensions outlined in the preceding Section II-B.

The obtained outcomes were compared to those of the standard low-pass birdcage coil. A phantom representing the volume-of-interest for *in ovo* imaging was used. The coils were loaded with the phantom (Ɛ = 80 and σ = 1.0 S/m) [34], tuned to 297.2 MHz, and matched to 50 Ω in the co-simulation, and driven in a quadrature mode with phase settings of 0 and 90°. The distance from the phantom to the coil at the centre was 5 mm. Once all the values of lumped elements were successfully determined, a final co-simulation was executed to derive the field distribution within the simulation domain [35]. The results of the transmit efficiency generated by the coils were analysed and compared. Here, the transmit efficiency (μT/√W) was normalised by the square root of the accepted power. The simulation data were utilised to pre-evaluate the proposed design prior to the construction of the actual coils.

### B. Modified Birdcage Coil Design and Construction

**Fig. 2.**
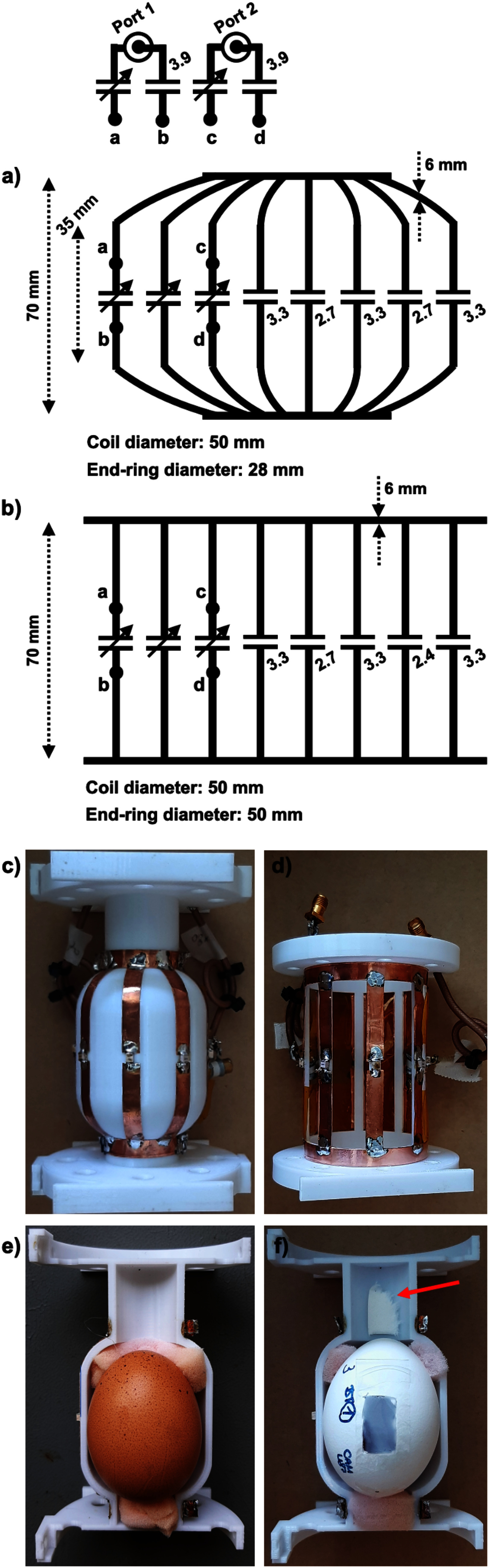
Schematic diagrams and photographs of the proposed modified (a, c) and conventional standard (b, d) birdcage coils, including essential component values (the unit of capacitors: pF) and geometrical information. Half-opened coils (e, f) are also presented to show the position of the eggs within the coil. The red arrow indicates the tissue for anaesthesia.

As displayed in Fig. 2(a) (schematic diagram) and (c) (photograph), a modified birdcage coil was designed and constructed. The coil includes a low-pass scheme operating in quadrature and a distinctive curved feature towards both end-rings.

The primary geometric specifications of the 8-rung birdcage coil are a length of 70 mm, a rung width of 6 mm and a diameter of 50 mm at its central region. The diameter gradually tapers down to 28 mm at the end-rings, commencing 17.5 mm away from the centre of the coil. In contrast to the standard coil, the modified coil almost maintains the distance between the object and the coil towards the end-rings. The geometry of the proposed coil was chosen based on the average size of ordinary chicken eggs. In order to assess the performance of the modified birdcage coil, we also built a conventional standard low-pass, quadrature birdcage coil shown in Fig. 2(b) and (d).

Coil formers were designed using 3D CAD software (Inventor, Autodesk, California, USA) and were 3D printed utilising an in-house 3D printer (Fortus 400mc, Stratasys, Minnesota, USA). The 3D printing material was biocompatible polycarbonate. This allowed us to shape the curved feature and to precisely duplicate thin but robust coil cases that minimised the sensitivity loss of the coil due to the filling factor. The geometry of the standard birdcage coil (70 mm in length and 50 mm in diameter) matched that of the proposed modified birdcage coil but did not incorporate the curved feature.

Having loaded eggs into the coils, both coils were tuned to 297.2 MHz (corresponding to the field strength of our 7 T MRI system) and matched to 50 Ω. The tuning and matching of the coils were accomplished using a combination of non-magnetic fixed and variable capacitors. Each point (a ∼ d) on the matching network is directly connected to the corresponding points (a ∼ d) on the coil.

In order to adjust the isolation between the two channels of the birdcage coils, we utilised a trimmer in the rung positioned between two feeding ports. The return losses (S_11_ and S_22_), isolation (S_21_) and Q-factors of these coils were measured using a vector network analyser (ZNB4, Rohde & Schwarz, Germany) on the bench for comparison.

Fig. 2(e) and (f) illustrate how the egg was positioned within the lower half of the modified coil case. A small window on the white eggshell in Fig. 2(f) was used for tumour implementation, and the piece of paper soaked in 2% isoflurane and placed above the egg delivered anaesthesia for the embryo.

### C. Experimental Preparation in Unfertilised Eggs and Chick Embryos

Unfertilised standard supermarket chicken eggs were used to generate ${{B}_1}^ + $ maps and to assess the SNR of the two coils. For the MR measurements using chick embryos, specific pathogen-free fertilised eggs suitable for medical purposes (Valo BioMedia GmbH, Germany) were also employed for *in ovo* MR measurements. These eggs were incubated at a temperature of 37.8°C with a humidity level of 54% in a benchtop incubator (HEKA Favorit-Olymp 192, Germany), starting from embryo development day 1.

On day five, a window in the eggshell was created, which allowed for the subsequent implantation of brain tumour cells. This was carried out using the glioma cell line U87-MG (ATCC HTB-14TM) that had been cultured in a standard cell culture medium. On day ten, the tumour cells were carefully introduced within a silicone ring placed on the CAM of the developing chick embryos. Following this procedure, the egg window was sealed with adhesive tape, and all eggs were returned to the incubator to facilitate tumour growth. When the embryos reached day 18, three eggs were used for MR measurements.

In order to ensure minimal movement during the MR measurements, the chick embryos were anaesthetised by pipetting a calculated amount of liquid isoflurane onto a piece of tissue paper (indicated by a red arrow) to reach 2% isoflurane within the confined airspace of one side of the RF coil former, as illustrated in Fig. 2(f). This step was vital to avoid image degradation due to excessive movement of the embryo.

### D. MR Measurements

All MR measurements were conducted on a 7 T Terra MRI scanner (Siemens Healthineers, Erlangen, Germany).

An actual flip-angle imaging (AFI) method [36], [37], [38] was employed in order to generate actual ${{B}_1}^ + $ maps of the proposed and standard birdcage coils. A supermarket egg was used for the measurements, and the key sequence parameters were repetition-time (TR) = 1000 ms; echo-time (TE) = 2.04, 6.12 and 10.20 ms; resolution = 1.2 × 1.2 × 7 mm^3^; flip angle = 30°; RF pulse type / duration = rectangular / 400 μs; number of averages (NEX) = 1; 3D acquisition and scan time = 8:31 minutes.

Furthermore, MR images of two supermarket eggs were obtained in the sagittal plane using a 2D turbo spin-echo sequence [39] with the following scan parameters: TR = 4200 ms; TE = 65 ms; in-plane resolution = 0.1 × 0.1 mm^2^; slice thickness = 1 mm; 30 slices; NEX = 2 and scan time = 3:59 minutes. Slightly different-sized supermarket eggs were chosen to guarantee access to more uniformly structured regions within the eggs for the SNR comparison between the coils. Four regions-of-interest (ROI) were selected, as indicated in Fig. 5, and the relative SNRs were calculated using the equation of the signal mean value (within the red dotted circles) divided by the noise standard deviation (S.D.) value (within the yellow dotted circles). Here, the noise images were acquired with zero transmit power.

Ultimately, the feasibility and applicability were demonstrated with three different chick embryos. For imaging the three chick embryos, a 2D gradient-echo sequence [40] was employed with the following acquisition parameters: TR = 15 ms; TE = 3.78 ms; flip angle = 25°; in-plane resolution = 0.1 × 0.1 mm^2^; slice thickness = 1 mm; 22 slices; NEX = 4 and scan time = 3:38 minutes. Due to the relatively complicated and inconsistent inner structures of the embryos, these embryo images were not suitable for coil comparison. Therefore, only the modified coil was used to obtain the embryo MR images.

## III. Results

**Fig. 3.**
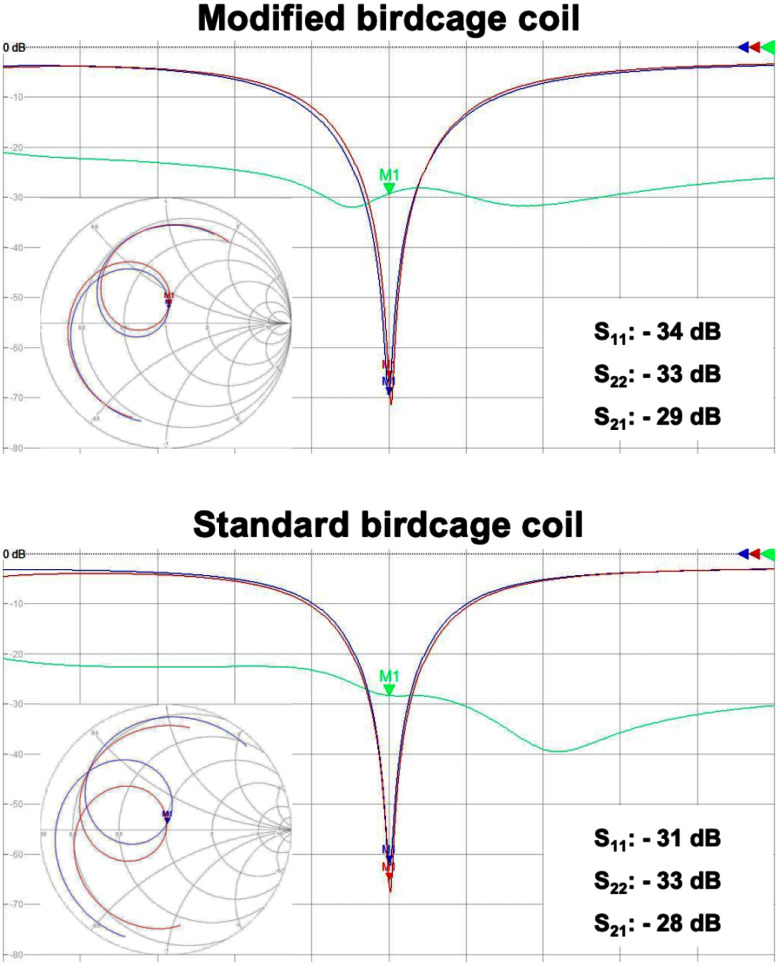
Screenshots of the responses of the modified and standard birdcages coils in dB and Smith Chart formats, obtained using the network analyser. Here, S_11_, S_22_ and S_21_ are depicted in red, blue and green, respectively.

The bench test results of the coils are shown in Fig. 3 and Table I. In Fig. 3, the return losses of all channels and the isolations between the channels of both coils were found to be better than -30 dB and -28 dB, respectively. This indicates an excellent tuning and matching condition of all coils to their resonance frequency and further demonstrates outstanding isolation between the quadrature ports of each coil. Furthermore, Table II includes the measured Q-factors, indicating that the proposed coil exhibits a lower loaded Q, which is attributable to its closer fit to the egg shape. Consequently, it demonstrates a higher Q-ratio.

**TABLE I:** Q-Factors (Unloaded/Loaded/Ratio) of the Coils

	Modified birdcage coil	Standard birdcage coil
Q_Unloaded_	208	217
Q_Loaded_	35	44
Q_Ratio_	5.9	4.9

**TABLE II:** Comparison of the SNR Values (Signal Mean/Noise S.D., Unit: a.u.)

	Modified birdcage coil	Standard birdcage coil
a	1629/5.8 =281	927/4.5 = 206
b	287/5.8 = 50	238/4.5 = 53
c	1625/5.7 = 286	1398/4.6 = 304
d	1800/6.1 = 290	1007/4.5 = 224
e	1310/4.9 = 267	824/4.7 =175
f	1315/4.9 =268	1289/4.7 = 274
g	242/4.8 = 50	217/4.7 = 47
h	1424/5.0 = 285	1152/4.7 = 245

Note: ‘a’ to ‘h’ refer to the ROIs in Fig. 3.

The modified birdcage coil was evaluated through simulation and was subsequently compared with the standard birdcage coil. The top row in Fig. 1 shows 3D simulation models of both coils, which were created based on the design structure and dimensions of the actual coils. The second row in Fig. 1 displays simulated ${{B}_1}^ + $ distributions of the phantom in the sagittal and the axial imaging planes for the modified birdcage coil (left column) and the standard birdcage coil (right column). The white dotted straight lines drawn in the sagittal image delineate the selected axial slices, where the head of the chick embryo may be located. As indicated, the modified coil exhibits comparable performance to the standard one in the central area, with less than a 5% difference, particularly in terms of ${{B}_1}^ + $ efficiency. However, as the imaging slice extends towards the end-rings, the modified coil demonstrates approximately 30% higher ${{B}_1}^ + $ efficiency compared with the standard coil. Additionally, the cross-sectional profiles displayed in Fig. 1(bottom row) show that the modified coil yields a more uniform ${{B}_1}^ + $ field across the designated region denoted as ‘a’ to ‘b’.

**Fig. 4.**
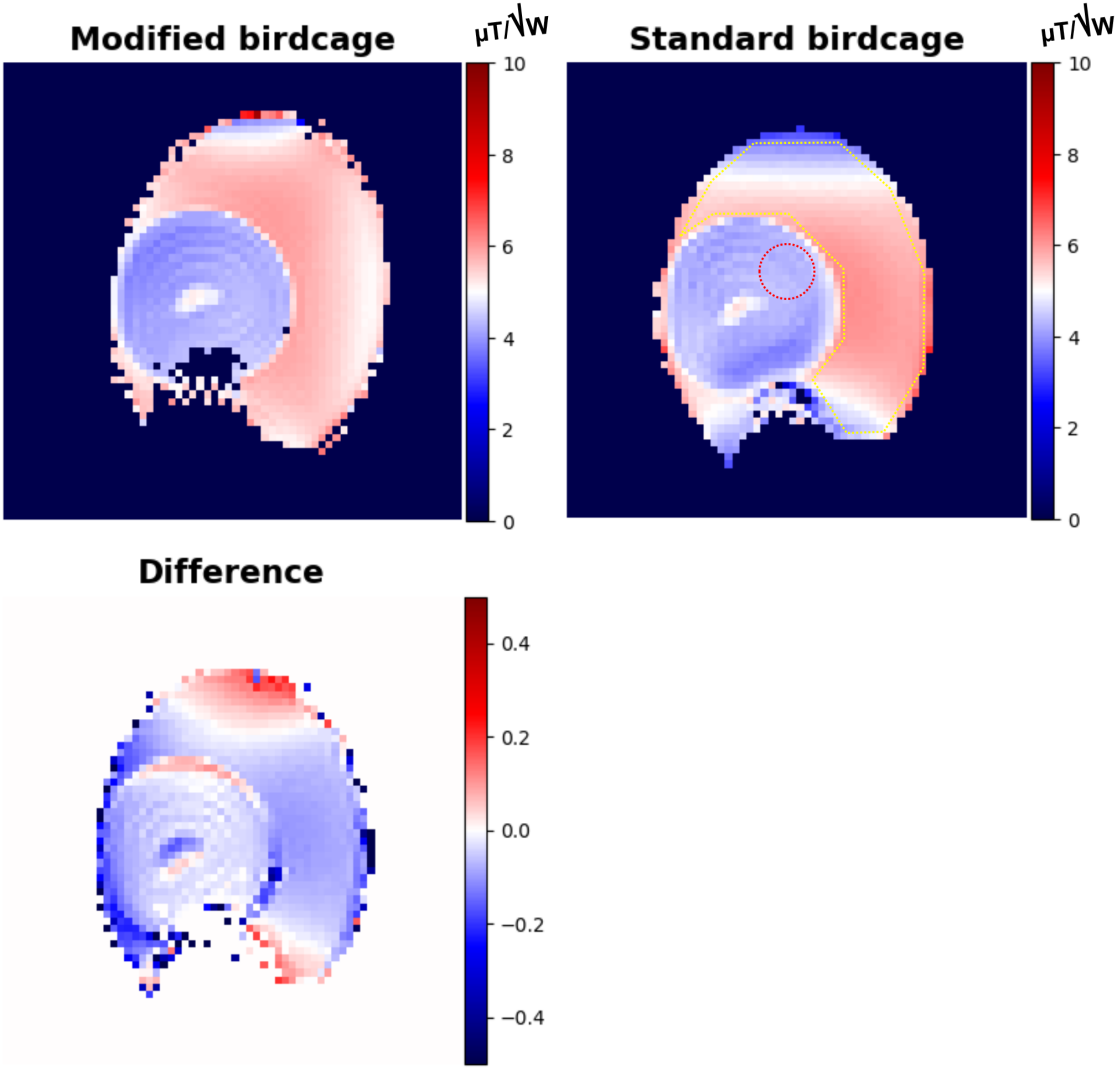
Measured ${{B}_1}^ + $ maps of the modified and standard birdcage coils obtained utilising AFI, and their difference map. The difference map was generated by subtracting the standard birdcage coil map normalised by the maximum value of μT/√W from the modified birdcage coil map normalised by the maximum value of μT/√W.

**Fig. 5.**
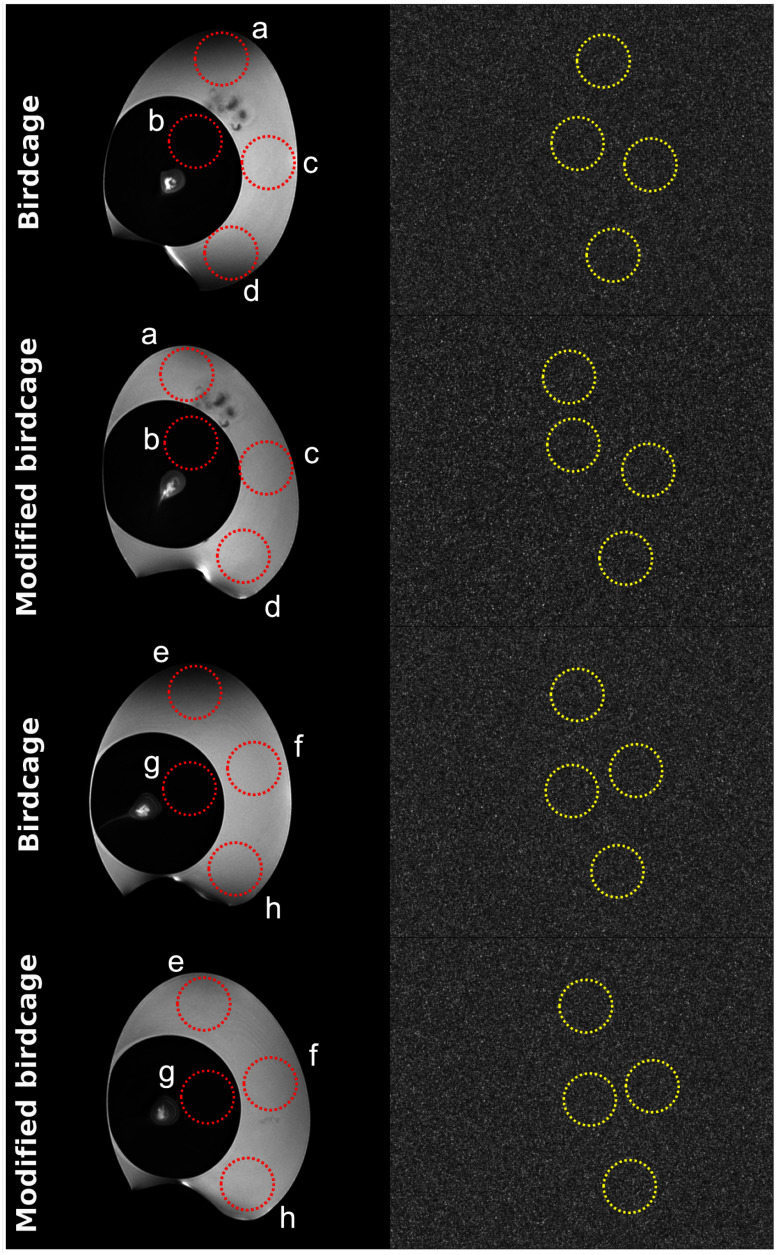
The MR images of supermarket eggs acquired using both birdcage coils, with corresponding noise images displayed on the right. The red and yellow dotted circles within the images represent various selected ROIs and the values in these regions are used for the calculation of SNRs.

Fig. 4 shows measured ${{B}_1}^ + $ maps of the modified and standard birdcage coils using AFI, along with their difference map. The observed trend of the ${{B}_1}^ + $ distribution in both coils aligns with the simulations in Fig. 1 and the imaging results in Fig. 5. The difference map was generated by subtracting the normalised standard birdcage coil map from the normalised modified birdcage coil map. Moreover, the mean and S.D. values of efficiency were calculated within the ROIs displayed in the standard coil map in Fig. 4. Specifically, within the egg yolk, these values were found to be 4.05 ± 0.14 μT/√W and 4.23 ± 0.12 μT/√W for the modified coil and the standard coil, respectively. However, within the egg white, they amounted to 5.56 ± 0.38 μT/√W and 5.43 ± 0.57 μT/√W, obtained using the modified and the standard birdcage coils, respectively.

Fig. 5 depicts sagittal images of supermarket eggs obtained using the modified and standard birdcage coils. In order to minimise potential bias stemming from egg position and size, we scanned two eggs of different sizes. Noise information was obtained from the images on the right collected without the application of transmit power. Various ROIs (‘a’ ∼ ‘d’ from one egg and ‘e’ ∼ ‘h’ from the other egg) were drawn in the egg white and egg yolk area. In these images, the ROIs highlighted in red represent signal regions, while those in yellow denote noise regions. Based on the selected ROIs given in Fig. 5, the associated SNR values of both coils are documented in Table II. Notably, the SNRs in the central swathes (ROIs ‘b’ and ‘c’ or ‘f’ and ‘g’) are nearly identical, with an average difference of less than 4%. However, in areas proximate to the end-rings (ROIs ‘a’ and ‘d’ or ‘e’ and ‘h’), there is a noteworthy increase in SNR in the images captured with the modified birdcage coil. The SNR increase varies from 16% (ROI: ‘h’) to 53% (ROI: ‘e’), with an average increase of 35%. When comparing the regions ‘a’, ‘d’ or ‘e’, it becomes evident that substantial signal degradation is observed in the standard birdcage coil, whereas the issue is less distinctive in the images acquired using the modified coil. When comparing the SNR values (mean ± S.D.) within the ROIs ‘a’, ‘c’ and ‘d’, we observed values of 245 ± 52.2 for the standard coil and 286 ± 4.5 for the modified coil. Additionally, in the ROIs ‘e’, ‘f’ and ‘h’, the calculated SNRs were 231 ± 50.9 and 273 ± 10.1 for the standard coil and the modified coil, respectively. This discrepancy emphasises the improved SNR and uniformity achieved near the end-rings with the proposed modified coil. This improvement is also evident in both the simulation results and the ${{B}_1}^ + $ maps depicted in Figs. 1 and 4.

**Fig. 6.**
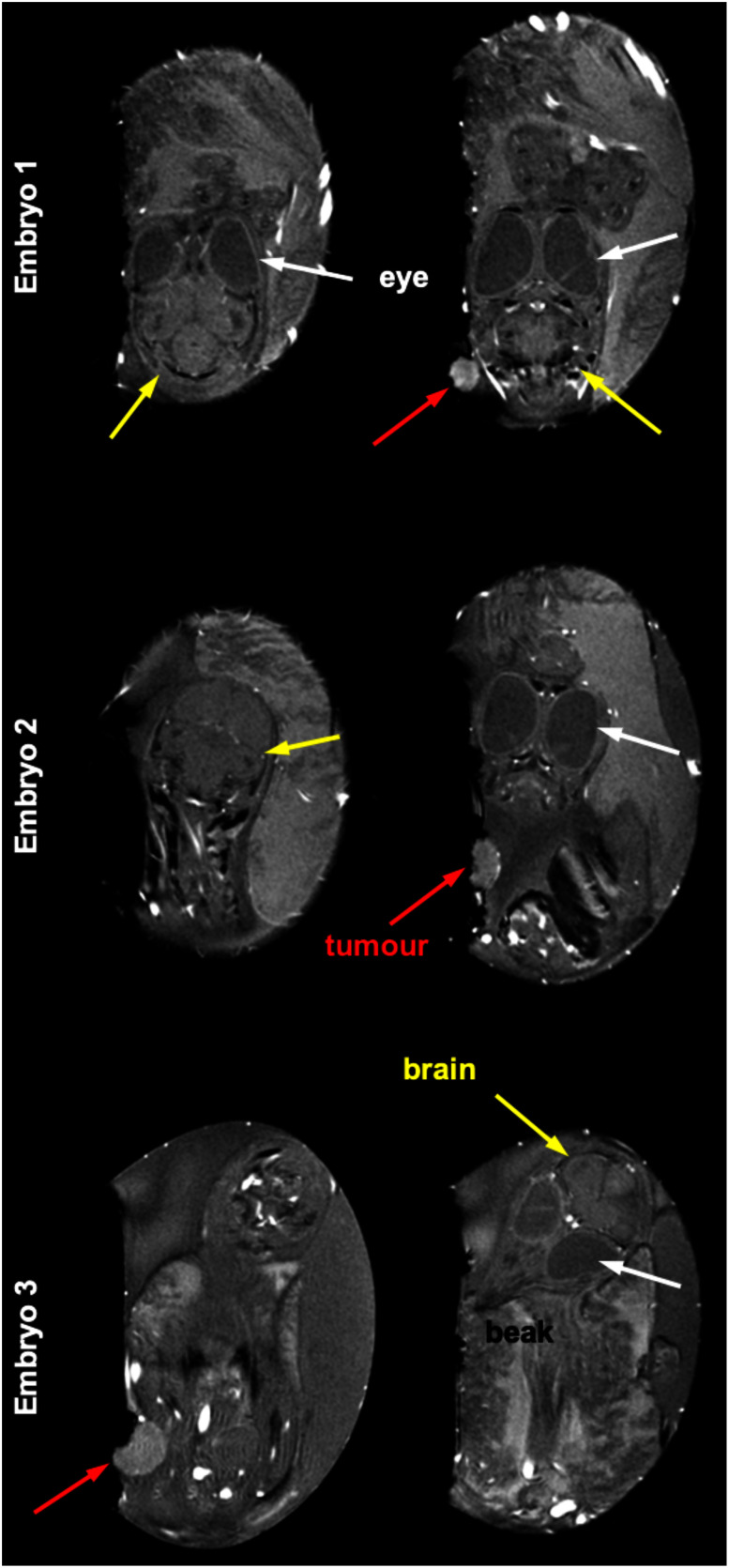
MR images of three different chick embryos obtained only using the proposed coil. Notable variation in head and brain positions can be seen - one centrally located in the eggshell and the others in the corners. These structures are also visible at different slices along the sagittal plane. Anatomical structures of chick embryos, such as eyes (indicated by white arrows), brains (indicated by yellow arrows), and beaks are identifiable. The presence of tumours on the chorioallantoic membrane is also highlighted with red arrows.

Fig. 6 shows MR images in different sagittal slices of three distinct chick embryos to demonstrate the feasibility of using the modified birdcage coil for real application. The positioning of each chick embryo's head and brain differs substantially. One is centrally positioned within the eggshell, while the others are situated in the extremities. Within the figure, various anatomical structures and body parts of the chick embryos are identifiable, which include the eyes (pointed out by white arrows), the brain (highlighted with yellow arrows), and the beak. Furthermore, the presence of tumours located on the CAM is apparent and these are clearly indicated by the red arrows.

## IV. Discussion

*In ovo* MR measurements represent a promising approach that can serve as a viable alternative to traditional *in vivo* preclinical MR experiments using small animals, as chick embryo brain tumours appear to have kinetics similar to those of rat brain tumours [4]. By utilising chick embryos, researchers can carry out ethical and expedited examinations using various methodologies.

The design concept of a modified birdcage coil for imaging chick embryos has been successfully demonstrated on a 7 T MRI scanner and has been proven to outperform the standard birdcage coil used for comparison. Whereas the SNR and ${{B}_1}^ + $ homogeneity of the standard birdcage coil was shown to significantly be degraded in the area close to the end-rings [12], [27], [28], the proposed coil design incorporates a curved feature which tapers down towards the end-rings in both directions, thus enhancing the transmit efficiency, the filling factor and the SNR. Here, the higher SNR was achieved with the modified coil, which has a higher filling factor and is more sample dominant (i.e., 20% higher Q-ratio shown in Table I), despite possessing slightly higher intrinsic loss (i.e., lower unloaded Q-factor, less than a 5% difference in Table I). The lower unloaded Q-factor of the modified coil may result from the extended rung length due to the curved feature, compared to the standard coil. Although we did not construct a longer standard coil, it may be an interesting comparison in the future as this would provide better ${{B}_1}^ + $ coverage across the egg in the z-direction, but without the tapered design (i.e., improving the filling factor), it is most likely that the SNR of the longer coil would be lower. Moreover, the modified coil ensures a broader and more consistent ${{B}_1}^ + $ field across the designated imaging area compared to the standard straight birdcage coil. These improvements are particularly crucial, as the positioning of a target body part of the chick embryo, e.g., a brain, can vary randomly, as shown in Fig. 6. Consistent coil sensitivity, regardless of the position, may be crucial when conducting longitudinal studies involving a substantial number of eggs, as there is typically no need for egg repositioning.

Further optimisation associated with the end-ring diameter may improve the coil sensitivity at both edges of the coil, albeit with a trade-off resulting in decreased ${{B}_1}^ + $ efficiency and SNR, to some extent, in the central region, as shown in Figs. 1 and 5. With our current design, the degradation at the periphery region of the centre slice was negligible in a simulated ${{B}_1}^ + $ profile (0.8%) and 4.3 ± 0.29% in SNR. Achieving complete closure of the end-rings might not be practically feasible, considering the requirement for space, particularly for coil construction and for anaesthesia purposes. In light of this, it may also be useful to explore alternative approaches, such as integrating a detachable structure [41] or a cap [42], [43], [44] in conjunction with the coil or using smaller end-rings.

The precise values of the electrical properties were not meticulously considered in the simulations, as the primary objective of the simulations was to understand the impact of incorporating the curved feature. Furthermore, since both designs were loaded with the identical phantom and tuned to this load, the simulations remain a valid comparison.

While our work was carried out on a clinical scanner, a small animal scanner would be more suitable for conducting similar studies. In such cases, the impact of the coil shield should also be carefully considered.

Using a multi-channel phased array coil combined with an independent transmit coil [45], [46] would also be an option, as it could potentially provide some SNR improvement, especially in the region nearest the coil. However, it may not be deemed necessary due to its complexity, high construction cost and the possible prerequisite for additional hardware units, such as a PIN-diode driver [47], [48]. Moreover, the target regions, e.g., an implanted tumour in the CAM or the brain, may not always be located within the highly sensitive region of the array coil at the time of measurement.

In general, a substantial number of *in vivo* small animals are required for most preclinical studies, and this is time-consuming. In order to address the issue of throughput and to accelerate the measurement efficiency, Bock et al. introduced a multiple coil approach [49]. Their work demonstrated the efficacy of carrying out simultaneous group measurements, thus considerably reducing the overall measurement time. This multiple coil scheme can also be applied to our proposed design for the imaging of chick embryos and would significantly expedite the measurement process for a large cohort of chick embryos. Moreover, as the chick embryos do not require preparation time or monitoring during the MR measurements, throughput would be further enhanced compared to small animal imaging experiments.

In this study, several limitations were encountered. One key constraint is the need to desolder in order to position the egg in the modified coil. This necessity arises from the prototype nature of the present coil; however, it could potentially be addressed by employing a connectorised system, which is commonly seen in human coils. For example, non-magnetic male (212-PINM-NM, Allectra GmbH, Germany) and female (212-PINF-NM, Allectra GmbH, Germany) crimp pins may be a suitable option for our coil. With this method, users would not need to physically change any parts of the coil, streaming the processes as simple as that using conventional coils. Another potential limitation is the variation in capacitor values inserted in each rung, which were not exactly identical but alternated due to the discretion and tolerance of the available capacitor values. Additionally, the applicability of specific sequences for particular applications warrants further investigation in future studies. Nonetheless, while these limitations are unlikely to significantly impact the overall performance of the coils, the improvements observed as a result of their implementation are highly likely to be valid.

## V. Conclusion

In ovo MR measurements using chick embryo models offer a promising and viable alternative with which to replace conventional in vivo preclinical MR experiments involving small animals. This approach is favoured for its straightforward preparation and numerous advantageous features. In this study, we have shown clear MR images of implanted tumours on the CAM of chick embryos. The innovative design of our proposed birdcage coil, distinguished by its unique curvature, provides more uniform ${{B}_1}^ + $ field from one extremity of the egg across to the opposite extremity with improved SNR compared to the standard coil. Thus, it has the potential to significantly enhance the quality of in ovo MR measurements.

## Conflicts of Interest

The authors declare no financial interests related to the subject of the manuscript.

## Authors’ Contributions

Study conception: CHC; System design: CHC; Hardware construction: MB; Hardware construction support: SS; EM simulation: SMH; Data acquisition: CHC, JF, MB, WW, SK; Object handling: SK, CS; Data analysis: CHC, JF, SMH; Supervision: CHC, NJS; Manuscript writing: CHC; Manuscript revision: All authors.
